# Exposure of *in vitro* maturing pig oocytes to exogenous interleukin-6 is associated with transcriptomic differences in the resulting blastocysts, with enrichment of developmental signaling pathways

**DOI:** 10.3389/fvets.2025.1716588

**Published:** 2026-01-05

**Authors:** Manuela Garcia-Canovas, Adelina Lopez-Jara, Inmaculada Parrilla, Alejandro Gonzalez-Plaza, Heriberto Rodriguez-Martinez, Maria A. Gil, Cristina Cuello, Emilio A. Martinez

**Affiliations:** 1 Department of Animal Medicine and Surgery, International Excellence Campus for Higher Education and Research “Campus Mare Nostrum” University of Murcia, Murcia, Spain; 2 Institute for Biomedical Research of Murcia (IMIB-Pascual Parrilla), Murcia, Spain; 3 Department of Biomedical & Clinical Sciences (BKV), BKH/Obstetrics & Gynaecology, Faculty of Medicine and Health Sciences, Linköping University, Linköping, Sweden

**Keywords:** IL-6, cytokine, *in vitro* embryo production, transcriptome, swine

## Abstract

**Introduction:**

Interleukin-6 (IL-6) has recently emerged as a promising supplement for *in vitro* maturation (IVM), enhancing pig blastocyst development and reducing apoptosis following *in vitro* fertilization (IVF). This study assessed whether IL-6 supplementation during IVM is associated with differences in gene expression in day-7 *in vitro*-produced blastocysts.

**Methods:**

Oocytes from prepubertal gilts were matured in IVM medium with (IL-6 IVM group) or without (control group) 100 ng/mL IL-6 and subsequently subjected to IVF. The resulting presumptive zygotes were cultured *in vitro* for 7 days. Six pools of seven viable blastocysts per group were flash-frozen in liquid nitrogen and stored at −80 °C until microarray analysis. The embryos were analyzed using the GeneChip® Porcine Genome Array (Affymetrix). Differentially expressed genes (DEGs) were identified by ANOVA using a significance threshold of *p* < 0.01 and a fold change > |1.5|.

**Results:**

Compared to the control, the IL-6 IVM group showed 91 up-regulated and 47 down-regulated DEGs. KEGG analysis of up-regulated genes revealed enrichment in 13 pathways including genes involved in key processes related to lipid metabolism, embryo development, cell proliferation, and immune system. Only two pathways were enriched among down-regulated genes, comprising genes not previously associated with essential roles in embryonic development.

**Discussion:**

Blastocysts derived from oocytes matured *in vitro* with 100 ng/mL of IL-6 exhibited differences in activity in several immune-related and lipid metabolism pathways through the overexpression of specific genes associated with cell proliferation, embryo development, and apoptosis reduction. These findings provide insights consistent with a role of IL-6 during porcine IVM and its association with oocyte developmental competence.

## 1 Introduction

The development of an efficient protocol of *in vitro* production (IVP) of pig embryos has significant implications for the advancement of reproductive biotechnologies. Traditionally, IVP protocols have included undefined biological substances, such as fetal bovine serum (FBS) or bovine serum albumin (BSA), whose variable composition can affect reproducibility in both *in vitro* maturation (IVM) and *in vitro* culture (IVC) stages. However, due to the uncertain composition and the eventual risks entailed, chemically defined media are preferred. In this context, cytokines have emerged as key supplements due to their crucial role in regulating physiological processes such as ovulation, embryonic development, and implantation. Particularly, interleukin-6 (IL-6) plays a pivotal role in regulating the ovarian follicular microenvironment. IL-6 has been shown to stimulate cumulus expansion by up-regulating the expression of *hyaluronan synthase 2* and *prostaglandin-endoperoxide synthase 2*, two key genes involved in extracellular matrix synthesis and cumulus expansion (1). Furthermore, IL-6 promotes nuclear maturation of the oocyte by activating multiple intracellular signaling pathways, including the JAK/STAT3 and MAPK cascades (1, 2). The incorporation of cytokines (individually or in combination) into IVP systems has been shown to enhance both the developmental competence and the quality of embryos in several species (3). Recent evidence indicates that IL-6 supplementation may modulate oocyte competence and subsequent embryonic development in pigs. The addition of 100 ng/mL IL-6 to the IVM medium has been shown to enhance oocyte quality, as evidenced by improved chromosome alignment and meiotic spindle conformation following maturation. Moreover, IL-6 supplementation during IVM was associated with higher developmental potential following IVF, resulting in blastocysts with increased total number of cells (TCN) and reduced apoptotic cell ratios. In contrast, IL-6 addition exclusively during IVC phase had no detectable effect, while combined supplementation during both IVM and IVC yielded outcomes similar to those obtained when supplementation was limited to IVM (4). These findings could imply a fundamental role of IL-6 in regulating the developmental competence of IVF-pig oocytes. This hypothesis is supported by two key observations. Firstly, IL6 transcripts have been recently identified as one of the most abundantly expressed cytokines in the bovine oviduct and endometrium at days 3 and 5 post-estrus (5). Secondly, significantly higher concentrations of IL-6 have been detected in endometrial tissue from inseminated pregnant sows compared to non-inseminated counterparts, supporting its potential immunoregulatory role in the establishment of pregnancy and in mediating maternal–embryonic dialog during the early stages of gestation (6). In mammalian embryos, the mechanism through which IL-6 exerts its effects remains complex and is not completely understood. Elucidating the mechanisms behind how IL-6 supplementation during IVM would affect developmental oocyte competence may improve our knowledge about how embryos adapt to the IVP system. The objective of this study was to improve porcine IVP outcomes by investigating whether exposure of oocytes to IL-6 (100 ng/mL) during IVM is associated with alterations detectable in day-7 blastocysts, without aiming to establish a direct causal effect on oocyte maturation. Interleukin-6 was applied exclusively during the maturation phase, which might enhance nuclear maturation through the classical IL-6R/gp130 signaling pathways (JAK/STAT3 and MAPK) with potential downstream effects on subsequent embryonic development.

## 2 Materials and methods

### 2.1 Chemicals and culture media

All chemicals used in the experiments were purchased, unless specified otherwise, from Sigma-Aldrich Química S. A. (Madrid, Spain). Detailed descriptions on the preparation of IVP media are available in (7). Briefly, a saline solution with 0.9 mg/mL NaCl and 70 mg/mL kanamycin was used for ovary transportation. Cumulus–oocyte complexes (COCs) were retrieved in Tyrode’s lactate medium supplemented with 0.1 mg/mL PVA and 10 mM HEPES (TL-HEPES-PVA). The IVM medium consisted of Tissue culture medium199 (Gibco Life Technologies S. A., Barcelona, Spain) enriched with 0.55 mM glucose, 0.9 mM sodium pyruvate, 75.0 mg/mL penicillin, 50.0 mg/mL streptomycin, 0.1 mg/mL PVA, 0.57 mM cysteine, and 10.0 ng/mL epidermal growth factor. Oocytes were stripped of cumulus cells using TL-HEPES-PVA with 0.1 mg/mL hyaluronidase. Semen samples were washed with Dulbecco’s phosphate-buffered solution containing 4 mg/mL BSA (DPBS-BSA). For *in vitro* fertilization (IVF), a Tris-buffered medium containing 0.3 mg/mL BSA and 2 mM caffeine was used. The embryo IVC medium consisted of North Carolina State University (NCSU)-23 medium, without BSA, supplemented with 0.3 mg/mL PVA and 100 ng/mL platelet factor-4.

### 2.2 *In vitro* embryo production

#### 2.2.1 Collection of cumulus–oocyte complexes

Pig ovaries were obtained from prepubertal crossbred gilts slaughtered at a local slaughterhouse (El Pozo S. A., Murcia, Spain) and transported to the laboratory within 2 h in saline solution at 35–37 °C. Immediately after arrival, the ovaries were washed, and COCs were collected in TL-HEPES-PVA at 38 °C, by sectioning the surface of medium-size follicles (3–6 mm) using a scalpel.

#### 2.2.2 *In vitro* maturation

Only COCs with multiple compact cumulus cell layers and granulated, homogeneous cytoplasm were used. The COCs were placed in groups of 70–80 in a four-well multidish plate (Nunc, Roskilde, Denmark), with 500 μL of IVM medium in each well. The IVM medium was previously equilibrated and supplemented with 10 IU of eCG (Folligon, Intervet International B. V., Boxxmeer, the Netherlands) and 10 IU of hCG (VeterinCorion, Divasa Farmavic, S. A., Barcelona, Spain) for the first 22 h of IVM. For the following 22 h of IVM, the COCs remained in the same medium without hormone supplementation. The incubations were performed at 38.5 °C under a paraffin oil overlay in a humidified atmosphere with 5% CO_2_ (8).

#### 2.2.3 *In vitro* fertilization

The COCs were denuded by vortexing at 1660 rounds/min for 2 min in 300 μL of TL-HEPES-PVA containing 0.1 mg/mL hyaluronidase. After denudation, the oocytes were washed three times in pre-equilibrated IVM medium, followed by three washes in pre-equilibrated IVF medium. Only oocytes exhibiting an intact zona pellucida (ZP) and a homogeneous cytoplasm were selected for IVF. Then, groups of 40 oocytes were placed in 50 μL drops of pre-equilibrated IVF medium, covered with mineral oil, and incubated for 30 min before the IVF. The semen straws used were previously cryopreserved with a high pre-freezing sperm dilution following the protocol described in (9). For each replicate, three straws were thawed in a 37 °C circulating water bath for 20 s. Thereafter, the spermatozoa were washed three times by centrifugation in Dulbecco’s phosphate-buffered solution containing 4 mg/mL BSA (1900xg for 3 min) and immediately resuspended in IVF medium. Subsequently, 50 μL of the sperm suspension was added to each drop containing oocytes (exposure of 2000 spermatozoa: oocyte). The coincubation period was carried out at 39 °C in atmosphere of 5% CO_2_ for 5 h.

#### 2.2.4 Culture of embryos

At the end of gamete coincubation, the putative zygotes were mechanically and repeatedly pipetted twice to eliminate spermatozoa attached to the ZP in drops of 500 μL of IVC medium. Zygotes were maintained in four-well dishes, in groups of 40–50, containing glucose-free culture medium with 0.3 mM sodium pyruvate and 4.5 mM lactate during the first 2 days. For the subsequent 5 days, they were transferred to the IVC medium supplemented with 5.5 mM glucose, instead of sodium pyruvate and lactate.

### 2.3 Preparation of samples and microarray hybridization

Sample preparation and microarray hybridization were performed as previously described (10). Total RNA was extracted from the day-7 blastocysts using a RNeasy Micro Kit (catalog number 74004; Qiagen Iberica, Madrid, Spain), and the quantity was measured by using a Nanodrop 2000 (ThermoFisher Scientific, Madrid, Spain). A GeneChip 3® IVT Pico Reagent kit (P/N 902790; Affymetrix, ThermoFiosher Scientific, Madrid, Spain) was used to synthesize single-stranded complementary DNA (sscDNA) from 650 pg. of RNA from each sample. The double-stranded DNA (dsDNA) targets were then purified, fragmented, and terminally labeled. A total of 4.5 μg of fragmented, biotin-labeled ds-DNA was incorporated into a hybridization mixture provided in the GeneChip Hybridization, Wash, and Stain Kit (catalog number 90720; Affymetrix), following the manufacturer’s instructions. The prepared samples were hybridized to the GeneChip® Porcine Genome Array (catalog number 900624, Affymetrix), designed to detect 20,201 genes and provide extensive coverage of the *Sus scrofa* transcriptome. After scanning the microarray chip, we performed data analysis using the Affymetrix Expression Command Console. All samples met the necessary quality standards.

### 2.4 Microarray data analysis

The intensity data from each GeneChip® array were normalized by using the robust multiarray assay (RMA) method (single-step background correction, quantile normalization, and log2 transformation), as implemented in Partek (Partek Incorporated, St. Louis, MS, United States). Partek Genomics Suite and Partek Pathways software were used for statistical analysis and biological interpretation of the data. We performed a principal component analysis (PCA) to assess the distribution of the datasets. Statistical analysis was based on ANOVA using treatment (IL-6 IVM vs. control) as the single experimental factor. An unadjusted *p*-value threshold of < 0.01 and fold change cutoff values of <−1.5 and >1.5 were applied to determine the significance of differentially expressed genes (DEGs). The same software was used to conduct pathway enrichment analysis for gene lists, with overrepresented pathways identified through the Kyoto Encyclopedia of Genes and Genomes (KEGG) database serving as a reference. Pathways with an enrichment score greater than 3 and a *p*-value below 0.05 were deemed to be enriched. The biological functions of genes potentially relevant to embryonic development and quality following IL-6 treatment during IVM were investigated using the DAVID database (Database for Annotation, Visualization, and Integrated Discovery).

### 2.5 Quantitative real-time PCR analysis

RNA extracted from the same samples was used for validation by real-time quantitative polymerase chain reaction (RT-qPCR) analysis. Maxima H Minus First Strand cDNA Synthesis Kit (Thermo Fisher Scientific) was used to transcribe RNA to cDNA. The primers (Table 1) were designed with Primer Express™ software v3.0.1 (Applied Biosystems, Foster City, CA, United States) and subsequently synthesized by a commercial provider. qPCRs analysis was performed using 10-μL of iTaqTM Universal SYBR Green Supermix containing 500 nM of each primer set. QuantStudioTM 5 Real-Time PCR system was used to perform all the reactions. Melting curve analysis was conducted to confirm the specificity of each PCR reaction, which was done by identifying a single peak in the dissociation curve profile. Additional samples were used in prior tests to determine the amplification efficiency of each primer pair, calculated with the formula E = 10^(−1/slope)^. Relative mRNA expression levels were determined using the Pfaffl method (11). Peptidylprolyl isomerase A (PPIA) and beta-actin (ACTB) served as reference genes for data normalization (12). Amplification efficiency for each target was also determined with the same formula.

**Table 1:** Primer sequences used for real-time quantitative PCR (RT-qPCR) analysis.

Gene symbol	Accession number		Primer sequences (5′-3′)	Size (pb)
*ACSL3*	NM_001143698	Forward	ATACCCTGGATGTGATACGCT	180
		Reverse	TGACCCAACATCTGTAAGCCA	
*FOS*	NM_001123113	Forward	CTACGAGGCGTCATCATCCC	181
		Reverse	GGTCGAGATAGCAGTCACCG	
*MAPK14*	XM_001929490	Forward	TGACCCAGATGCCGAAGATG	122
		Reverse	GGCTTGGGCTGCTGTAATTC	
*PPP2CA*	NM_214366	Forward	AGAGGTTCGATGTCCAGTCAC	200
		Reverse	TGGTGATGCGTTCACGGT	
*SLC9A3*	XM_013992324	Forward	GCACCATCCTCCTCTATGCC	169
		Reverse	ACGATGATGAAGAGCACCTCG	
PPIA*	XM_021078519.1	Forward	CTGAAGCATACGGGTCCTGG	100
		Reverse	CCAACCACTCAGTCTTGGCA	
GAPDH*	NM_001206359.1	Forward	ATCACTGCCACCCAGAAGAC	194
		Reverse	AGATCCACAACCGACACGTT	

*Housekeeping genes.

### 2.6 Experimental design

Oocytes from prepubertal gilts were collected and matured in IVM medium either supplemented with 100 ng/mL IL-6 or without supplementation (IL-6 IVM and control groups, respectively), with IL-6 added directly into the IVM medium and maintained throughout maturation, and subsequently fertilized *in vitro*. Putative zygotes from each group were cultured for 7 days in IVC medium, after which blastocysts were selected for analysis. These blastocysts were derived from the same experimental replicates as those used for morphological and apoptosis assessment in the prior study (4) where oocytes from the IVM + group exhibited higher blastocyst formation efficiency, greater total cell numbers, and reduced apoptosis compared with controls. Six pools of seven viable embryos (*n* = 42 embryos per group) were placed in sterile Eppendorf, immersed in N2, and stored at −80 °C until microarray analysis. After microarray analysis, a list of DEGs was generated for comparison of the treatment group and the control. Five genes (four up-regulated genes and one down-regulated gene, according to the microarray results) were selected to perform the RT-qPCR for microarray validation.

### 2.7 Statistical analysis

Microarray analyses included six biological replicates per group (each replicate a pool of seven blastocysts). PCA and hierarchical clustering were conducted on all arrays (*n* = 12). The Shapiro–Wilk test was applied to evaluate the normality of the RT-PCR data, which followed a parametric distribution. RT-qPCR data were analyzed by Student’s t-test with Levene’s test for homogeneity; significance was set at *p* < 0.05.

## 3 Results

In the same experimental dataset we previously reported (4), IL-6 supplementation during IVM did not alter cleavage rates but significantly increased blastocyst efficiency (38.2 ± 5.22% vs. 29.3 ± 5.2%; *p* < 0.05), yielding blastocysts with higher total cell numbers and fewer apoptotic cells (both *p* < 0.05) compared with controls.

**Figure 1:**
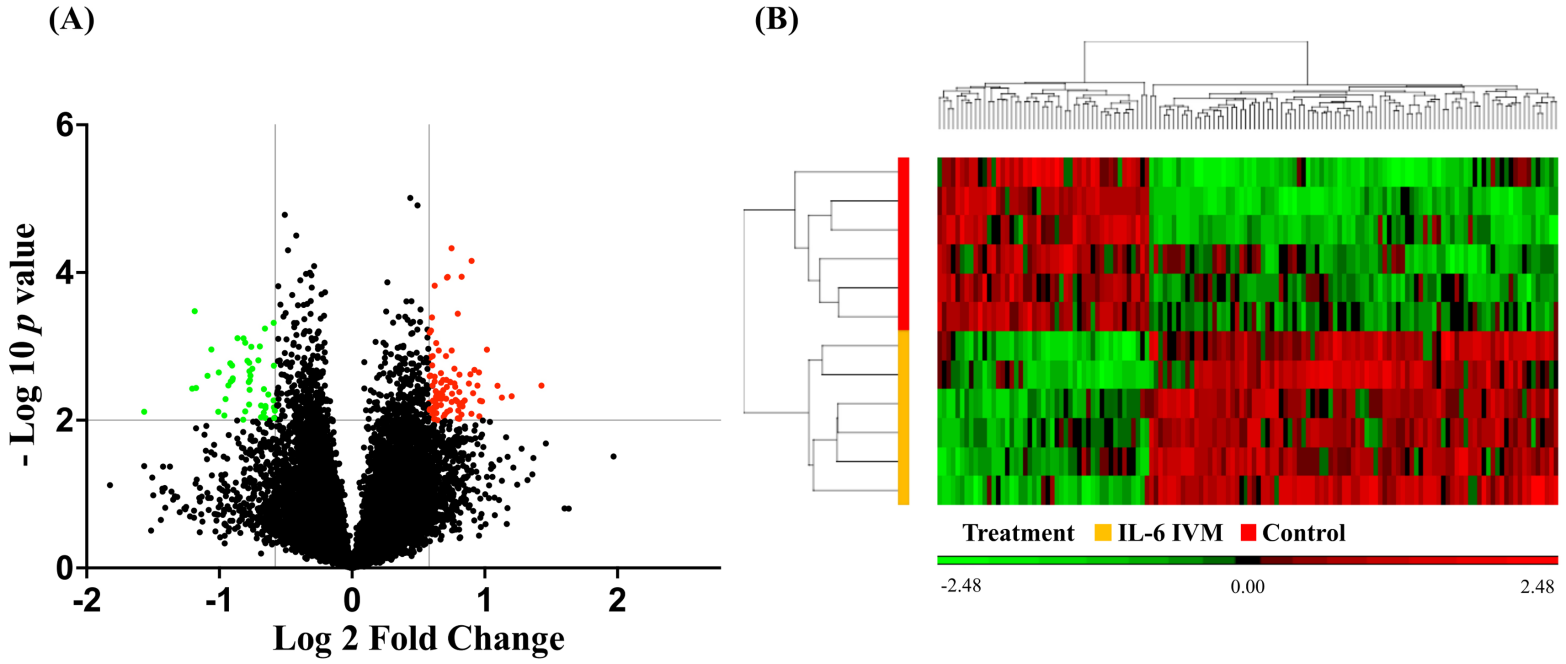
Volcano plot **(A)** and heat map **(B)** comparing differentially expressed genes between blastocysts derived from oocytes treated with IL-6 during IVM (IL-6 IVM) and non-treated oocytes (control group). In the volcano plot, the color indicates different levels of gene expression (Red: up-regulation; green: down-regulation; black: genes that did not meet the significance thresholds (unadjusted *p*-value of < 0.01 and fold change of <−1.5 and >1.5). In the heat map, color indicates expression levels of DEGs (red: up-regulation; green: down-regulation).

Treatment with exogenous IL-6 during IVM of oocytes was associated with significant differences in the transcriptome profile of porcine embryos. The PCA analysis successfully clustered IL-6-treated IVM samples separately from control samples based on all gene expression data, revealing 50.8% of variance between both groups, with the first axis explaining 21.5%. Analysis of the transcriptome profile of the blastocysts revealed 16,462 annotated transcripts, which were subjected to subsequent analysis. A total of 138 transcripts, annotated as known genes, were differentially expressed in embryos from the IL-6 IVM group compared to controls (Supplementary Table 1). Of these, 91 were up-regulated and 47 were down-regulated. The volcano plot represents the DEGs detected in blastocysts from IL-6 IVM group compared to the control group (Figure 1). Figure 1 also shows the results of the hierarchical cluster analysis, which clearly distinguishes the IL-6 IVM samples from the control samples.

### 3.1 GO terms and pathway enrichment of DEGs

**Figure 2:**
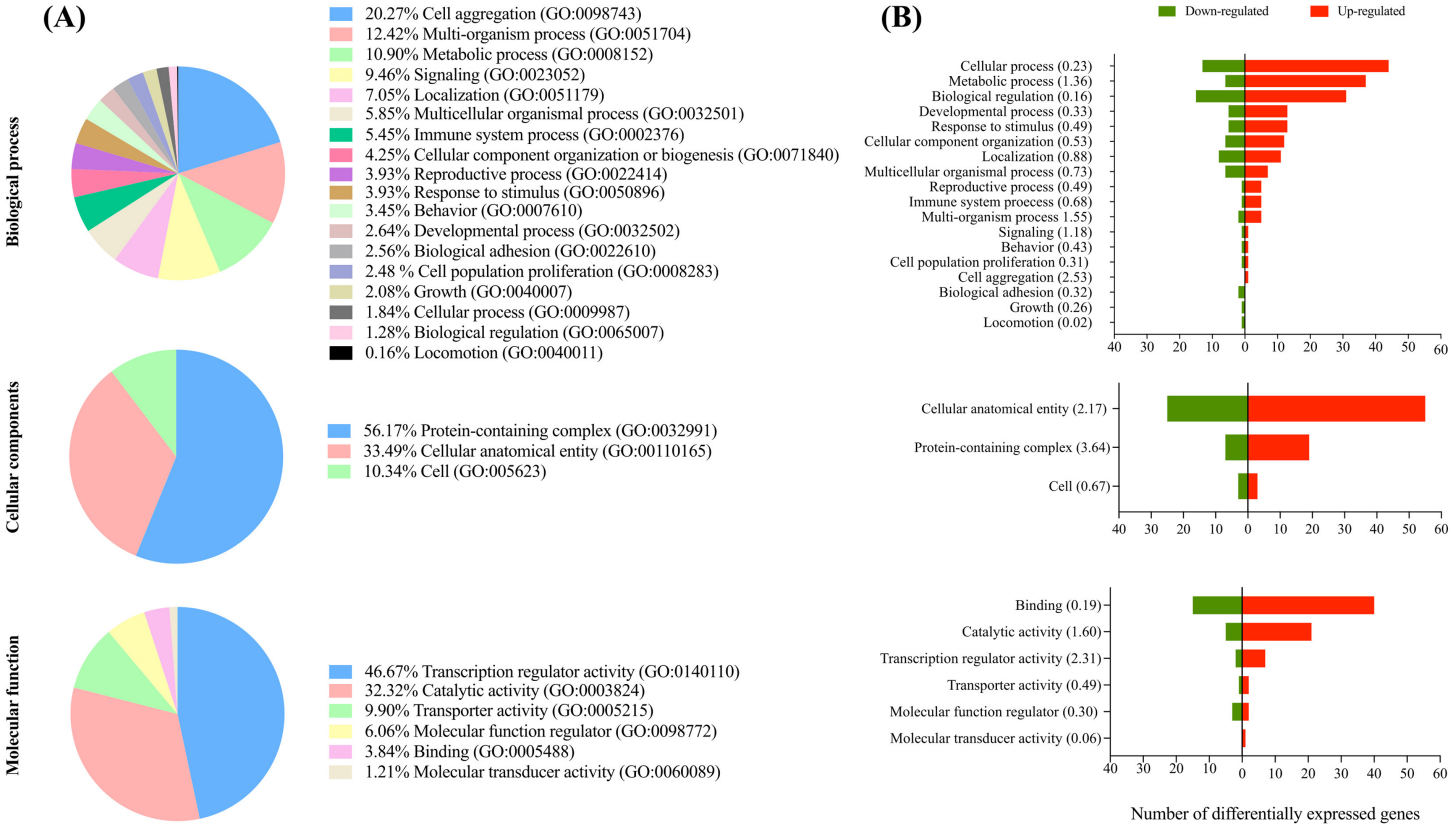
Functional enrichment analysis. **(A)** Gene Ontology analysis of the differentially expressed genes (DEGs) between embryos derived from oocytes treated with IL-6 during IVM and control group; percentages indicate the proportion of DEGs assigned to each category, and the parentheses show the corresponding GO term IDs. **(B)** For the same set of GO categories summarized in panel A, bar plots display the number of up- and down-regulated genes per term. Values in parentheses are enrichment scores. The x-axis represents the absolute number of DEGs.

The GO terms analysis classified the DE transcripts into the categories of biological processes, cellular components, and molecular functions, according to the KEGG database. These three principal categories were subsequently subdivided into second-level GO terms (Figure 2). The terms with the highest enrichment score in the category of biological process included cell aggregation, multi-organism process, and metabolic process. Within cellular components, the category of protein-containing processes and cellular anatomical entities were the most highly represented. In the molecular functions category, the most enriched terms were transcription regulator activity, catalytic activity, and transporter activity.

**Figure 3:**
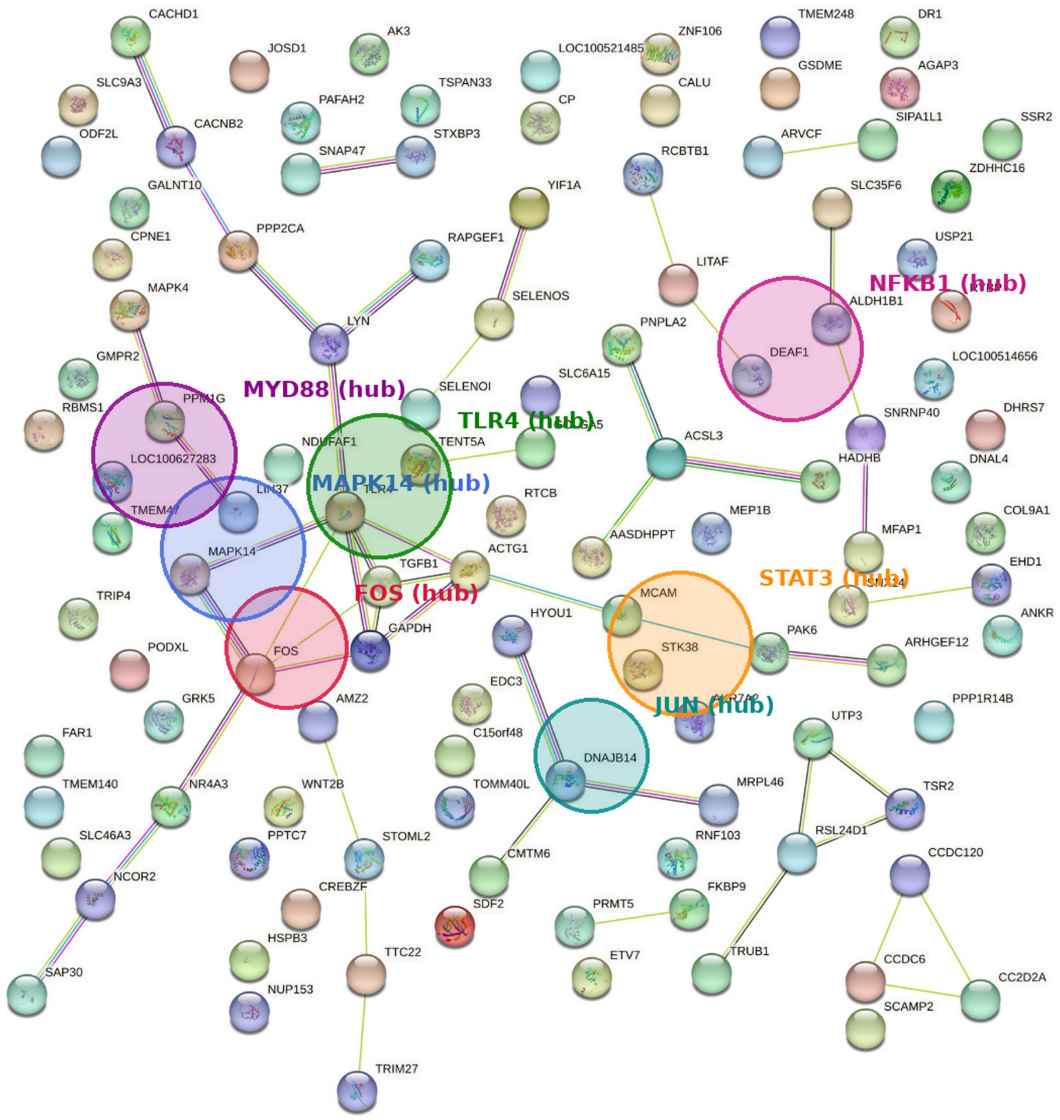
Functional interaction network among proteins encoded by differentially expressed genes (DEGs) between blastocysts derived from oocytes treated with IL-6 during IVM and control group was obtained with the String database and Gene Ontology classification. The nodes represent DEGs. Edges represent the predicted functional associations. An edge may be drawn with up to four different colored lines, and these lines represent the existing associations that were predicted. A green line: neighborhood evidence; a blue line: cooccurrence evidence; a purple line: experimental evidence; a yellow line: text-mining evidence; a black line: co-expression evidence. Hub nodes are highlighted with halos. Primary hubs: FOS, MAPK14, TLR4. Additional IL-6-linked hubs: STAT3, MYD88, JUN, NFKB1. Together these hubs connect innate immune signaling to stress-activated MAPK and AP-1/NF-κB transcriptional responses, consistent with a role for IL-6 as an upstream coordinator during oocyte maturation.

Dataset lists of overall, upregulated, and down-regulated DEGs were used to determine those significantly enriched KEGG pathways (Table 2). We observed enrichment in 8, 13, and 2 pathways in the overall, upregulated, and down-regulated lists, respectively, resulting in a total of 15 pathways that were specifically affected by the treatment. KEGG pathways analysis in the upregulated list showed enrichment of pathways associated with fatty acid degradation, ether lipid metabolism, IL-17 signaling pathway, toll-like receptor signaling pathway, T cell receptor signaling pathway, platelet activation, phosphonate and phosphinate metabolism, Th17 cell differentiation, relaxin signaling pathway, signaling pathways regulating pluripotency of stem cells, sphingolipid signaling pathway, hippo signaling pathway, and valine, leucine and isoleucine degradation pathway. These pathways included genes as *ACSL3, ALDH1B1, HADHB, EPT1, PAFAH2, FOS, MAPK4, MAPK14, TLR4, PAK6, LYN, ARHGEF12, SMAD2, WNT2B, PPP2CA, and SPTLC1*, many of which are involved in key lipid metabolism, embryo development, and immune system-related processes. Only two pathways were enriched by down-regulated genes: focal adhesion and protein digestion and absorption, and included genes as *ACTG1, COL9A1, RAPGEF1, and SLC9A3* with a key role in adhesion and absorption functions (Table 3). Figure 3 presents the functional interaction network of proteins encoded by the genes differentially expressed between IL-6-treated and control samples, obtained using the STRING database and classified according to Gene Ontology annotations (13).

**Table 2:** Enrichment analysis of KEGG pathways in embryos derived from treatment with IL-6 IVM compared to control embryos.

Pathway ID	Pathway name	Enrichment *p*-value		
		All	Up	Down
ssc04390	Hippo signaling pathway	**0.008**	**0.049**	0.069
ssc00071	Fatty acid degradation	**0.012**	**0.004**	-
ssc04611	Platelet activation	**0.017**	**0.027**	-
ssc04659	Th17 cell differentiation	**0.024**	**0.035**	-
ssc04926	Relaxin signaling pathway	**0.024**	**0.035**	-
ssc00565	Ether lipid metabolism	**0.037**	**0.017**	-
ssc04974	Protein digestion and absorption	**0.041**	-	**0.028**
ssc00440	Phosphonate and phosphinate metabolism	**0.043**	**0.028**	-
ssc04657	IL-17 signaling pathway	0.056	**0.016**	-
ssc04620	Toll-like receptor signaling pathway	0.065	**0.022**	-
ssc04660	T cell receptor signaling pathway	0.070	**0.024**	-
ssc04550	Signaling pathways regulating pluripotency of stem cells	-	**0.036**	-
ssc04071	Sphingolipid signaling pathway	-	**0.040**	-
ssc00280	Valine, leucine, and isoleucine degradation	-	**0.048**	-
ssc04510	Focal adhesion	0.091	-	**0.020**

Enriched pathways with significant values in each gene list are shown in bold.

**Table 3:** Enriched (*p* < 0.05) KEGG pathways in embryos derived from treatment with IL-6 IVM and control embryos.

Pathway ID	Pathway name	Pathway alteration (expression)	ES	Altered genes (%)	Gene list
ssc00071	Fatty acid degradation	Over-	5.5	8.8	ACSL3, ALDH1B1, HADHB
ssc00565	Ether lipid metabolism	Over-	4.1	9.5	EPT1, PAFAH2
ssc04657	IL-17 signaling pathway	Over-	4.1	5.2	FOS, MAPK4, MAPK14
ssc04620	Toll-like receptor signaling pathway	Over	3.8	4.6	FOS, MAPK14, TLR4
ssc04660	T-cell receptor signaling pathway	Over-	3.7	4.5	FOS, MAPK14, PAK6
ssc04611	Platelet activation	Over-	3.6	4.3	MAPK14, LYN, ARHGEF12
ssc00440	Phosphonate and phosphinate metabolism	Over-	3.5	33.3	EPT1
ssc04659	Th17 cell differentiation	Over-	3.3	3.9	FOS, MAPK14, SMAD2
ssc04926	Relaxin signaling pathway	Over-	3.3	3.9	FOS, MAPK14, SMAD2
ssc04550	Signaling pathways regulating pluripotency of stem cells	Over-	3.3	3.9	MAPK14, SMAD2, WNT2B
ssc04071	Sphingolipid signaling pathway	Over-	3.2	3.7	MAPK14, PPP2CA, SPTLC1
ssc04390	Hippo signaling pathway	Over-	3.0	3.4	PPP2CA, SMAD2, WNT2B
ssc00280	Valine, leucine, and isoleucine degradation	Over-	3.0	5.4	ALDH1B1, HADHB
ssc04510	Focal adhesion	Under-	3.9	2.5	ACTG1, COL9A1, RAPGEF1
ssc04974	Protein digestion and absorption	Under-	3.5	3.7	COL9A1, SLC9A3

### 3.2 Validation of microarray results

**Figure 4:**
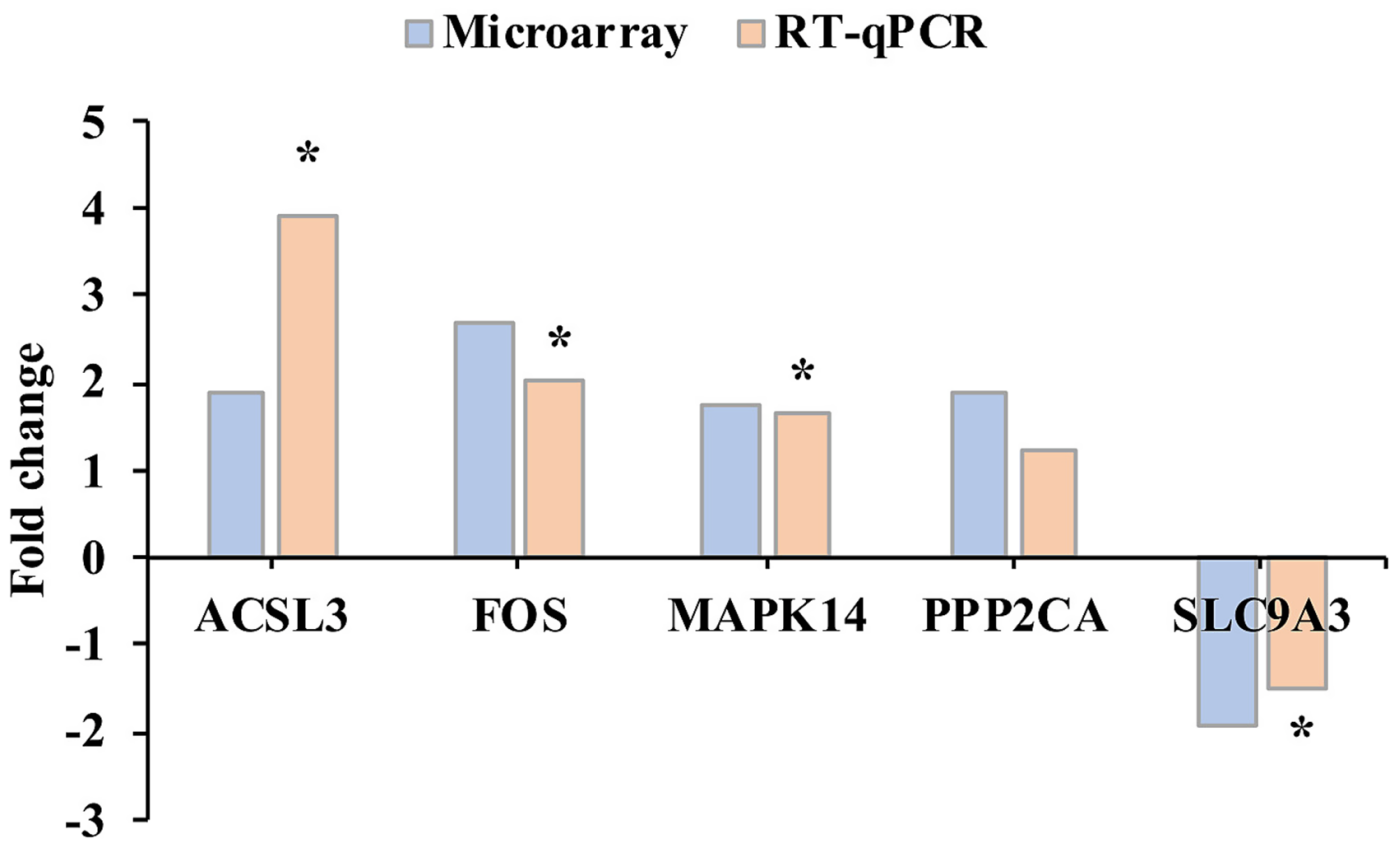
Validation of microarray results by real-time quantitative PCR (RT-qPCR). The Y-axis represents the log₂ fold change between the IL-6 IVM and control groups. Asterisks indicate significant differences between IL-6 IVM and control groups based on RT-qPCR analysis (Student’s *t*-test; *p* < 0.05). Data represent six biological replicates.

A total of five genes were used for validation of the microarray data using RT-qPCR. The expression trends observed by RT-qPCR were consistent with the fold-change patterns obtained from the microarray analysis (Figure 4), thereby supporting the reliability of the transcriptomic results.

## 4 Discussion

Here, we present the first analysis examining the association between exogenous IL-6 (100 ng/mL) supplementation during porcine IVM and the transcriptomic profiles of the resulting IVP blastocysts. The findings reveal differential activity in immune-related and lipid metabolism pathways, characterized by the overexpression of genes associated with cell proliferation, embryonic development, and reduced apoptosis, providing context for potential associations on blastocyst quality and developmental competence.

The GO term enrichment analysis of blastocysts from IL-6 IVM group revealed that most of the DEGs involved in biological process, cellular components, and molecular functions were up-regulated compared to control blastocysts. It is well established that the interaction of IL-6 with its receptor complex triggers multiple intracellular signaling cascades that regulate a broad range of cellular processes, including proliferation, differentiation, survival, lipid metabolism, and apoptosis (14). This aligns with the findings of our KEGG pathway-enrichment analysis. Specifically, analysis of DEGs revealed that IL-6 treatment was associated with differences in metabolic and immune-related signaling pathways. Among the up-regulated DEGs, 13 KEGG pathways were significantly enriched, including those regulating fatty acid degradation and ether lipid metabolism, both of which are central to lipid metabolism. Lipids play critical roles in various biological processes, such as cell growth, oocyte maturation, and embryonic development (15). Genes in these pathways include *ACSL3*, *ALDH1B1*, *HADHB*, *EPT1*, and *PAFAH2*. Notably, *ACSL3* is involved in the generation of long-chain acyl-CoA and regulates cellular fatty acids uptake in diverse cell types, including trophoblasts (16). Its overexpression may indicate increased fatty acid oxidation (17), a process essential for embryonic development (18). The sphingolipid signaling pathway was also altered by up-regulated genes, including *MAPK14*, *PPP2CA*, and *SPTLC1*. Sphingolipids are essential for maintaining cellular membrane structure and regulating diverse biological processes (19). *SPTLC1* is a catalytic subunit of the SPT complex, which is essential for sphingolipid biosynthesis. *SPTLC1* has been implicated in regulating gastrulation, and its knockout in mice results in embryonic lethality (20). Furthermore, *SPTLC1* is significantly up-regulated in mouse uterine stromal cells, suggesting a potential role in the decidualization process (21).

The IL-17 signaling and the Th17 cell differentiation pathways were also enriched by overexpressed genes, such as *FOS*, *MAPK4*, *MAPK14,* and SMAD2. The IL-17 family, predominantly produced by Th17 cells, comprises pro-inflammatory cytokines (IL-17 A-F) that regulate the immune system and are involved in female reproduction (22). Specifically, IL-17D appears to play a pivotal role in the development of embryos in murine (23) and porcine species, following somatic cell nuclear transfer (24) by regulating apoptosis-associated pathways. Additionally, the role of IL-17 in human trophoblast invasion and proliferation was investigated using the JEG-3 cell line as a model (25). The results indicated that IL-17 enhanced the invasive capacity of JEG-3 cells without affecting their proliferation or multinucleated cell formation, suggesting a regulatory role of IL-17 in human trophoblast invasion. Based on these findings, the enrichment of pathways related to Th17 cells and IL-17 production may influence invasion competence, cell proliferation, and potentially provide a protective effect on embryos derived from IL-6 IVM treatment.

Toll-like receptor and T-cell receptor signaling pathways are also related to the modulation of immune system and were enriched by overexpressed genes (*FOS*, *MAPK4*, *MAPK14*, and *TLR4*). Toll-like receptors are critical components of the innate immune system, producing cytokines and chemokines involved in reproductive processes (26). During pregnancy, *TLR4* activation is essential for both innate and adaptive immunity, protecting embryos from pathogens. A recent study demonstrated the overexpression of several proteins involved in TLR4 cascade pathways, including *MAPK14*, in the endometrium of pregnant sows during early gestation. This highlights the role of *TLR4* in the embryo-endometrium interaction and immune balance, which is crucial to establish a favorable environment for pregnancy (27).

The MAPK gene family is implicated in a variety of cellular processes, including proliferation, differentiation, apoptosis, and development (28). In particular, *MAPK14* (also known as p38 MAPK) is a key component of the p38 MAPK cascade and seems essential for preimplantation development in mice (29, 30). Inactivation of *MAPK14* has been linked to early embryonic lethality and placental morphogenesis defects (31, 32). *MAPK14* also plays a role in regulating filamentous actin during preimplantation development (33), and its deficiency has been associated with cytoskeletal disruption and cytokinesis failure in hepatocytes. Such disruption of actin filaments may affect the meiotic spindle formation as well, as proper cytoskeletal organization is essential for accurate chromosome alignment and segregation during cell division (34). The overexpression of *MAPK14* could be consistent with the improved meiotic spindle conformation observed in IL-6 IVM oocytes compared to the control group (4). Since *MAPK14* plays a critical role in maintaining actin cytoskeleton integrity, its enhanced activity may promote proper spindle assembly and chromosome alignment, essential for timed meiotic division and cell proliferation. Conversely, while *MAPK4* has been implicated in promoting cell survival and inhibiting apoptosis in certain contexts and cells, such as in glioma cells (35), its overall role in development appears less critical. *MAPK4*-deficient mice are viable, fertile, and exhibit no apparent physical or physiological anomalies (36). However, given the potential protective role of *MAPK4* in other cellular processes, it is plausible that its up-regulation in IL-6-treated IVM oocytes may contribute to improved embryonic development.

The hippo signaling pathway, a highly conserved pathway in mammals, plays a crucial role in modulating organ size, cell proliferation, apoptosis, and differentiation of trophectoderm (TE) cells and inner cell mass (ICM) (37). This pathway was enriched by genes such as *PPP2CA*, *SMAD2*, and *WNT2B*. A recent study revealed that lysophosphatidic acid (LPA), an agonist of the hippo signaling pathway, significantly improved embryo development and quality in terms of TCN and apoptosis of bovine embryos cultured *in vitro* (38). Similarly, LPA has been shown to enhance embryo development and quality in porcine embryos (39). Specifically, *PPP2CA* encodes the phosphatase 2α catalytic subunit, which plays an essential role in regulating cell growth and proliferation. In addition, the absence of *PPP2CA* in mice results in delayed embryonic lethality (40).

The *FOS* gene was overexpressed in several enriched pathways, including IL-17 signaling, Toll-like receptor signaling, T cell receptor signaling, Th17 cell differentiation, and relaxin signaling pathways. *FOS*, a proto-oncogene, is implicated in cell proliferation, differentiation, apoptosis regulation, and trophoblast migration and invasion (41). It is expressed in preimplantation blastocysts of mammals, including pigs (42). Our findings align well with previous studies (43) demonstrated that IL-6 supplementation in mouse embryo IVC medium counteracted the adverse effects of superovulation and altered *FOS* gene expression. Similarly, exogenous IL-6 supplementation was observed to promote cell proliferation and maintained pluripotency in mouse embryonic stem cells through *FOS* overexpression (44). These findings, taken together, suggest that the up-regulation of *FOS* may be associated with the enhanced cellular proliferation observed in embryos derived from IL-6 IVM treatment.

The SMAD gene family, which encodes signal transducers and transcriptional modulators modulating multiple signaling pathways related to cell proliferation, apoptosis, and differentiation, was overexpressed in several pathways, including Th17 cell differentiation, relaxin signaling, pluripotency of stem cells, regulating signaling pathways, and the Hippo signaling pathway. *SMAD2*, a member of this family, is widely tissue-expressed and plays a role in early embryonic development (45). Its deficiency is associated with significant disruptions in gastrulation and mesodermal differentiation (46). Our findings are consistent with published research demonstrating that seminal plasma infusions administered before artificial insemination promoted the overexpression of genes associated with embryonic development and implantation of *in vivo* porcine blastocysts, including *SMAD2* (47). Based on these findings, we suggest that *SMAD2* expression may serve as a potential biomarker for oocyte viability and developmental competence.

The KEGG pathways analysis revealed two pathways enriched by down-regulated genes: focal adhesion and protein digestion and absorption. Genes that altered the focal adhesion pathway were *ACTG1*, *COL9A1*, and *RAPGEF1*. This pathway is essential for regulating cell–extracellular matrix interactions and communication. In particular, the *ACTG1* gene encodes actins proteins, major components of the cytoskeleton involved in cell motility, division, signaling, and maintenance of cell shape. However, mice deficient in *ACTG1* resulted in viable embryos during embryonic development, suggesting that its activity may be compensated by other isoforms of actin (48, 49). However, its specific mechanisms and impacts on embryonic development remain unclear. *RAPGEF*, ubiquitously expressed in vertebrates, plays a pivotal role in multiple signaling pathways linked to cell differentiation and apoptosis. It is essential for fibroblast adhesion and spreading, as well as early murine embryonic development (50). While it can be up-regulated during myocyte differentiation and apoptosis (51), down-regulation has been linked to enhance migratory and stemness properties in liver oval cells (52). The specific impact of *RAPGEF1* on the pig embryo transcriptome requires further investigation. The other pathway enriched by down-regulated genes, protein digestion, and absorption, included genes, such as *COL9A1* and *SLC9A3,* which appear to have no direct association with reproductive processes.

Altogether, these molecular changes offer a mechanistic context for previously reported improvements in blastocyst yield, total cell number, and apoptosis after IL-6 supplementation during IVM and inform rational media optimization strategies.

The mechanistic basis for potential long-term effects of short-term IL-6 exposure during IVM lies in its ability to engage the receptor complex (IL6R/gp130), activating JAK kinases, STAT3, and MAPK pathways (14). During IVM, such signaling may induce molecular and metabolic changes that persist into preimplantation development. IL-6-mediated JAK/STAT and MAPK activity could influence cytoplasmic maturation by altering post-transcriptional control of maternal mRNAs or by modifying key proteins. Because these maternal factors regulate the maternal-to-zygotic transition, even brief alterations in their abundance or translation may affect early developmental outcomes. IL-6 can also modulate immunometabolic routes that support oocyte competence and embryo development (15–19). The observed up-regulation of *ACSL3, HADHB, ALDH1B1,* and *SPTLC1* suggests enhanced lipid metabolism and signaling, processes linked to energy balance, membrane remodeling, and cell-fate regulation. Moreover, IL-6 exposure may activate MAPK14/p38 and AP-1/FOS, lowering the activation threshold for stress- and differentiation-related responses during cleavage and blastocyst formation (28–30, 33, 41–43). In cumulus cells, IL-6 signaling may intersect with SMAD2-dependent pathways that influence cumulus expansion and paracrine support to the oocyte (45). Given the reliance on gap-junctional and paracrine communication, even short-term pathway modulation can alter the oocyte’s molecular state. In our study, although embryos were not exposed to IL-6 during IVF or IVC, IL-6-induced changes in maternal mRNA translation, protein modification, or metabolic regulation during IVM may persist beyond embryonic genome activation. These mechanisms could cause the KEGG pathway enrichments observed here (Hippo, pluripotency, immune signaling, and lipid metabolism) as downstream outcomes of IVM-stage reprogramming rather than direct cytokine exposure in culture. Taken together, these observations outline a mechanistic explanation consistent with our transcriptomic findings. Future studies should elucidate causal relationships through integrated transcriptomic and epigenetic profiling of oocytes and cumulus cells, coupled with imaging and tracer studies to visualize IL-6 uptake and assays to quantify lipid content in these cells.

## 5 Conclusion

Blastocysts derived from IVF pig oocytes matured in the presence of IL-6 are associated with differences in various immune-related and lipid metabolism pathways through the overexpression of specific genes associated with cell proliferation, embryo development, and apoptosis reduction. These findings provide insights consistent with a role of IL-6 during porcine IVM and its association with oocyte developmental competence, at least to the blastocyst stage, potentially providing crucial information for optimizing future IVM protocols.

## Data Availability

The datasets presented in this study can be found in online repositories. The names of the repository/repositories and accession number(s) can be found in the article/Supplementary material.

## Supplementary material

SUPPLEMENTARY TABLE 1 Differentially expressed genes in embryos derived from oocytes matured with IL-6 (IL-6 IVM group) compared to controls.
