# Correction: Exposure of *in vitro* maturing pig oocytes to exogenous interleukin-6 is associated with transcriptomic differences in the resulting blastocysts, with enrichment of developmental signaling pathways

**DOI:** 10.3389/fvets.2026.1802076

**Published:** 2026-02-24

**Authors:** Manuela Garcia-Canovas, Adelina Lopez-Jara, Inmaculada Parrilla, Alejandro Gonzalez-Plaza, Heriberto Rodriguez-Martinez, Maria A. Gil, Cristina Cuello, Emilio A. Martinez

**Affiliations:** 1 Department of Animal Medicine and Surgery, International Excellence Campus for Higher Education and Research “Campus Mare Nostrum” University of Murcia, Murcia, Spain; 2 Institute for Biomedical Research of Murcia (IMIB-Pascual Parrilla), Murcia, Spain; 3 Department of Biomedical & Clinical Sciences (BKV), BKH/Obstetrics & Gynaecology, Faculty of Medicine and Health Sciences, Linköping University, Linköping, Sweden

**Keywords:** IL-6, cytokine, *in vitro* embryo production, transcriptome, swine

In the published article, there was a mistake in the **Funding** statement.

The **Funding** information for the Key Development Project was displayed as “PID2022-138666OB-I00 funded by MICIU/AEI/10.13039/501100011033”. The correct information reads: “PID2022-138666OB-I00, funded by MICIU/AEI/10.13039/501100011033 and ERDF, UE”

The correct **Funding** statement reads:

